# Effect of energy drinks on the surface microhardness of bulk fill resins. In vitro study

**DOI:** 10.4317/jced.61896

**Published:** 2025-02-01

**Authors:** Jennifer Loo-Valle, Denisse Aguilar Gálvez, Luis Ernesto Arriola-Guillén

**Affiliations:** 1 Division of Pediatric Dentistry, School of Dentistry, Universidad Científica del Sur, Lima, Peru; 2 Division of Orthodontics, School of Dentistry, Universidad Científica del Sur, Lima, Peru

## Abstract

**Background:**

The study aimed to compare the surface changes of Filtek Z350XT, Filtek Bulk Fill, and Opus Bulk Fill resins immersed in Maltin Power® and Volt® energy drinks using surface microhardness testing.

**Material and Methods:**

This experimental study evaluated two types of bulk fill resins and Filtek resin (control). Forty-eight resin discs measuring 4x4 mm were prepared and soaked in 20 ml of an energy drink for 10 minutes daily for 7 and 30 days. Surface microhardness was assessed using the Vickers microhardness method. Repeated samples were analyzed using the ANOVA test, and multiple comparisons were conducted using the Bonferroni test. Finally, the Kruskal-Wallis test also was applied (*P*< 0.05).

**Results:**

The decrease in surface microhardness of the three resins exposed to the two energy drinks was significant (*P*<0.05). After immersion in the Volt energy drink over 30 days, the Filtek Z350XT, Filtek Bulk Fill, and Opus Bulk Fill resins showed decreases in surface microhardness of 3.89±1.94; 7.74±4.66 and 5.86±3.17, respectively, while the respective decreases after immersion in the Maltin Power energy drink were 3.67±4.08, 5.70±0.99, and 3.23±1.76.

**Conclusions:**

Immersion in the energy drinks changed the surface microhardness of bulk fill resins after 7 and 30 days. Both clinicians and patients should consider these findings when determining the consumption frequency of these beverages.

** Key words:**Energy drink, hardness test, composite resins.

## Introduction

The consumption of energy drinks among children and adolescents has risen due to the perceived benefits of heightened activity, physical endurance, reduced fatigue, and improved mental acuity (1,2). These drinks are composed of a large number of carbohydrates (sucrose, glucose), amino acids, such as taurine, proteins, B complex vitamins (B1, B2, B6, B12), vitamin C, niacin, pantothenic acid, and one of its main ingredients is caffeine (3). However, health problems, such as arrhythmias, insomnia, obesity, increased blood pressure, and even stroke, have been reported concerning the consumption of these drinks (4-7). Studies have also shown that high consumption of energy drinks causes dental erosion due to their degree of acidity and may also cause damage to the organic matrix of the resins (8-11).

In the last decade, a new generation of composite resins, called bulk fill resins, has emerged. These resins exhibit lower polymerization contraction, improved light penetration, and increased curing depth, facilitating increases of 4 to 5 mm (12).

The surface microhardness of composite resins is a crucial characteristic as it enables these resins to resist elastic and plastic deformation and damage when subjected to stresses from external sources. This microhardness is a critical factor to consider when evaluating restorative materials, determining their success, which can be affected by incomplete polymerization, inadequate polishing, dental erosion caused by carbonated substances or drinks, energizers, and moisture (13).

Microhardness tests measure the resistance of a material to penetration in indentations. This test is accomplished by applying a force to the material using an indenter and measuring the depth of the resulting penetration. This measurement provides a hardness value for the material (14), with a higher value in the microhardness tests providing greater penetration resistance. The best-known methods include the Vickers, Knoop, Rockwell, Brinell, and Shore durometry tests (15-19).

To our knowledge, there has been only one specific study on this topic (20) and more studies are needed to determine the changes in the surface of bulk fill resins when exposed to energy drinks. Therefore, the purpose of the present study was to compare the surface changes of Filtek Z350XT, Filtek Bulk Fill, and Opus Bulk Fill resins following immersion in Maltin Power® and Volt® energy drinks using surface microhardness as the measurement.

## Material and Methods

The current study was an experimental *in vitro* study approved by the Ethics Committee of Universidad Cientifica del Sur under registration code 502-2019-POS8. The selection criteria included using resin discs with precise measurements, smooth surfaces, and no bubbles or fractures. All resin discs were handled by a single operator who had received training in specimen preparation, including a pilot study for training and calibration. Three different composite resins from the Filtek Z350 brands were included. The sample size was determined based on a pilot test and previous research. Forty-eight resin discs were obtained, divided into six groups of 8 each. The control group consisted of Filtek Z350 XT resin discs.

-Preparation of resin discs

The resin discs were created using a prefabricated and standardized 3M brand matrix measuring 4 mm in diameter and 4 mm in height. The matrix was coated with liquid vaseline using a brush to facilitate easy removal of the composite resin discs. Subsequently, 3M Filtek Z350XT resin, 3M Filtek Bulk Fill resin, and Opus Bulk Fill FGM resin were applied using the Hu-Friedy Flex XTS resin Spatula. A layer of AIRON Maquira celluloid tape was placed over the resin, and a glass stage was used to apply pressure to the matrix and the resin.

After removing the glass stage, the material was cured using a Elipar™ DeepCure-S 3M lamp with a light intensity of 1470 mw/cm2. The curing method involved placing the lamp at a distance of 0 mm with a centered light guide (ISO 4049) on the celluloid matrix. The curing times were 20 seconds for a 2 mm thickness in the Filtek Z350 3M resin, 20 seconds for a 4 mm thickness in the Filtek Bulk Fill resin, and 30 seconds for a 4 mm thickness in the Opus Bulk Fill resin.

-Polishing of resin discs

After creating the resin discs, they were polished using a 3M Sof-Lex finishing and polishing disc system according to the manufacturer’s instructions. The resin discs were then randomly divided into three groups and labeled. Finally, the discs were placed in a beaker of distilled water, covered with aluminum foil to protect them from light, and stored in a Yamato oven at 37°C for 24 hours.

-Initial microhardness measurement

The resin surfaces were analyzed for initial microhardness in millinewtons (mN) using the Vickers microhardness method with a digital Vickers HV-1000 LG durometer. The equipment was pre-set to apply a 50g load over 15 seconds. Three separate indentations were made on the surface of each sample, spaced 100 micrometers apart.

-Exposure to energy drinks

After measuring the initial microhardness, the samples were immersed in 20 ml of each Maltin Power® and Volt® energy drink. The 48 resin discs were divided into six groups and soaked in the energy drinks for 10 minutes once a day for seven days and then for 30 days.

-Final microhardness measurement

Before the final test, the resin discs were washed with distilled water and dried with paper towels. Then, the final microhardness measurement was taken using the same parameters as the initial measurement.

-Statistical analysis

The results were stored in a Microsoft Excel database and analyzed with the Stata ® version 16.0 program. We calculated central tendency and dispersion measures, such as mean, standard deviation, and minimum and maximum surface microhardness values for each resin group. The data from the Filtek Bulk Fill group did not show a normal distribution, and thus, we used the Kruskal-Wallis Test for analysis. We used the ANOVA test for repeated samples to analyze surface microhardness at three different times. The significance level was set at *P*<0.05.

## Results

 Table 1 shows the comparison of the surface microhardness of the Filtek XT, Filtek Bulk Fill, and Opus Bulk resins following immersion in an energy drink for 10 minutes daily for seven and 30 days. The ANOVA and Friedman’s tests showed statistical differences in the microhardness of the different resins according to the time periods of immersion in the two drinks (*p*= 0.001). After immersion in the Volt energy drink over 30 days, the Filtek Z350XT, Filtek Bulk Fill and Opus Bulk Fill resins showed decreases in surface microhardness of 3.89±1.94; 7.74±4.66; and 5.86±3.17, respectively, while the respective decreases after immersion in the Maltin Power energy drink were 3.67±4.08, 5.70±0.99, and 3.23±1.76.

Additionally, the Kruskal Wallis test indicated no statistically significant differences in the decrease in surface microhardness of the three resins after immersion in either the Volt (*p*= 0.086) or the Maltin power drink (*p*= 0.298) ( Table 2).

## Discussion

Various studies have shown that the surfaces of resins can be altered by carbonated drinks (19–21), isotonic drinks (22,23) and energy drinks (24,25). In the present study, the effects of two energy drinks, Volt®, and Maltin Power®, on the surface microhardness of three resins were evaluated. The resins examined were Filtek XT, Filtek Bulk Fill, and Opus Bulk Fill. It was observed that the energy drinks caused alterations on the surface of the resins, which intensified over time. The results could be explained by the presence of components such as citric acid and its derivatives, (26) as well as the very low pH of the drinks (27).

We utilized Vickers microhardness testing because it can measure all types of hardness, especially with small thicknesses. Its wide range of test loads allows for versatile applications across various materials with high hardness. This method offers the advantage of using a single scale to cover a wide range of hardness and is non-destructive, allowing for the samples to be used after testing (13).

It is important to note that the erosive potential of beverages depends on a complex interplay of various factors, such as the type of acid, its concentration, the duration of time the drink stays in the oral cavity, and the buffer capacity of saliva (28). The current study results show that energy drinks such as Volt® (pH 3.81) and Maltin Power® (pH 4.25) have a high erosive potential due to their acidity, as indicated by their low pH values. This result supports the findings of Cavalcanti *et al*. (29). Acidity is one of the primary causes of dental erosion in children and adolescents, as evidenced by studies establishing a direct link between drink acidity and dental tissue loss (10,27).

The current study found no significant differences in the erosive potential between the two energy drinks. Al-Samadani (30) and Tanthanuch *et al*. (20) supported these findings, demonstrating that the reduction in surface hardness of restorations increased with an increase in immersion time. However, Erdemir *et al*. (23) found that one energy drink brand in their study had the highest erosive effect, with a pH value below 2.67. They suggested that a low pH could increase the diffusion coefficient in resins, water absorption, and solubility parameters, leading to accelerated degradation of the organic matrix, reduced surface microhardness, and shortened restoration lifespan (31).

On the other hand, the present study found no significant difference in the decrease in surface microhardness between the Filtek XT, Filtek Bulk Fill, and Opus Bulk Fill resins after immersion in energy drinks. This result is consistent with the findings of a previous study by Tanthanuch *et al*. (20) comparing the surface microhardness of a nanohybrid composite resin and a bulk fill resin, which also showed similar reductions in microhardness. Therefore, it is important to avoid the consumption of energy drinks because they have been proven to cause damage to composite resins. This research is significant because composite resins are available internationally. Several studies have demonstrated that exposure to acidic pH drinks, such as energy drinks, can affect the surface hardness of all composite resins (21,24,25,26,32,33). In this sense, our results help dentists and patients by providing appropriate recommendations on the frequency of consumption of these drinks to ensure the longevity of dental restorations.

## Conclusions

There was a noTable reduction in the surface microhardness of the resins evaluated after immersing the resin discs in Maltin Power® and Volt® energy drinks for 7 and 30 days. Clinicians should take these changes into account when using these resins.

## Figures and Tables

**Table 1:** Comparison of the surface microhardness of Filtek XT, Filtek Bulk Fill, and Opus Bulk resin when immersed in an energy drink at baseline for 10 minutes daily for seven and 30 days.

		Baseline Mean ± SD	7 Days Mean ± SD	30 Days Mean ± SD	p
Filtek Z350 XT	Volt	63.56±5.64a	61.10±4.99b	59.68±4.50b	0.001+
	Maltin Power	63.96±4.49a	61.34±4.76b	60.29±5.33c	0.001+
Filtek Bulk Fill	Volt	52.92±4.45a	48.41±5.33b	45.19±6.37c	0.001++
	Maltin Power	48.40±2.79a	44.49±3.01b	42.65±3.20b	0.001+
Opus Bulk Fill	Volt	35.55±3.37a	32.27±2.40b	29.69±2.91c	0.001+
	Maltin Power	35.14±3.76a	33.36±4.66b	31.91±4.74b	0.001+

(+) Anova test for repeated samples, the Bonferroni test was used for multiple comparisons. (++) Friedman test, for multiple comparisons and the Wilcoxon sign-rank test was used. Different lower-case letters indicate statistically significant difference in each row. SD: standard deviation

**Table 2:** Evaluation of the surface microhardness of composite resins immersed in Volt and Maltin Power energy drinks from baseline to day 30.

Energy drinks	Resins	Mean ± SD	P
Volt	Filtek Z350 XT	3.89±1.94	0.086+
	Filtek Bulk Fill	7.74±4.66	
	Opus Bulk Fill	5.86±3.17	
Maltin power	Filtek Z350 XT	3.67±4.08	0.298+
	Filtek Bulk Fill	5.70±0.99	
	Opus Bulk Fill	3.23±1.76	

(+) Kruskal Wallis test. SD: standard deviation

## Data Availability

The datasets used and/or analyzed during the current study are available from the corresponding author.
